# A novel nomogram model combining CT texture features and urine energy metabolism to differentiate single benign from malignant pulmonary nodule

**DOI:** 10.3389/fonc.2022.1035307

**Published:** 2022-12-15

**Authors:** Jing Shen, Hai Du, Yadong Wang, Lina Du, Dong Yang, Lingwei Wang, Ruiping Zhu, Xiaohui Zhang, Jianlin Wu

**Affiliations:** ^1^ Graduate School, Tianjin Medical University, Tianjin, China; ^2^ Department of Radiology, Affiliated Zhongshan Hospital of Dalian University, Dalian, China; ^3^ Department of Radiology, Ordos Central Hospital, Ordos Inner Mongolia, China; ^4^ School of Medicine, Dalian University, Dalian, China; ^5^ Department of Research, Dalian Detecsen Biomedical Co., LTD, Dalian, China; ^6^ Graduate School, Dalian Medical University, Dalian, China; ^7^ Graduate School, Dalian University, Dalian, China; ^8^ Department of Cardio-Thoracic Surgery, Affiliated Zhongshan Hospital of Dalian University, Dalian, China; ^9^ Department of Pathology, Affiliated Zhongshan Hospital of Dalian University, Dalian, China; ^10^ College of Environment and Chemical Engineering, Dalian University, Dalian, China

**Keywords:** pulmonary nodules, nomogram, texture analysis, urine energy metabolism, benign, malignant

## Abstract

**Objective:**

To investigate a novel diagnostic model for benign and malignant pulmonary nodule diagnosis based on radiomic and clinical features, including urine energy metabolism index.

**Methods:**

A total of 107 pulmonary nodules were prospectively recruited and pathologically confirmed as malignant in 86 cases and benign in 21 cases. A chest CT scan and urine energy metabolism test were performed in all cases. A nomogram model was established in combination with radiomic and clinical features, including urine energy metabolism levels. The nomogram model was compared with the radiomic model and the clinical feature model alone to test its diagnostic validity, and receiver operating characteristic (ROC) curves were plotted to assess diagnostic validity.

**Results:**

The nomogram was established using a logistic regression algorithm to combine radiomic features and clinical characteristics including urine energy metabolism results. The predictive performance of the nomogram was evaluated using the area under the ROC and calibration curve, which showed the best performance, area under the curve (AUC) = 0.982, 95% CI = 0.940–1.000, compared to clinical and radiomic models in the testing cohort. The clinical benefit of the model was assessed using the decision curve analysis (DCA) and using the nomogram for benign and malignant pulmonary nodules, and preoperative prediction of benign and malignant pulmonary nodules using nomograms showed better clinical benefit.

**Conclusion:**

This study shows that a coupled model combining CT imaging features and clinical features (including urine energy metabolism) in combination with the nomogram model has higher diagnostic performance than the radiomic and clinical models alone, suggesting that the combination of both methods is more advantageous in identifying benign and malignant pulmonary nodules.

## Introduction

Lung cancer is one of the malignant tumors with high morbidity and mortality, the incidence and mortality rates of which have been on the rise in recent years. The incidence and mortality of lung cancer in China ranked first among all malignant tumors in 2015 with those in the world at about 11.4% and 18%, respectively (1). The 5-year survival rate can approach 50% if early diagnosis and therapy are successful, and early detection and treatment of lung cancer are the keys to improving patient survival rates (2). With the wide application of high-resolution CT, pulmonary nodules are ubiquitous in CT screening. Benign and malignant pulmonary nodules have different treatments and prognoses. Semantic characteristics of pulmonary nodules such as size, attenuation, and margins are often insufficient for characterization. Follow-up CT increases the cost and radiation burden on the patient, in addition to the patient’s concern about waiting too long to learn the results (3–5). Therefore, the accurate diagnosis of lung nodules is particularly important. Although conventional CT features are helpful in identifying benign and malignant nodules, there is still some controversy as to which morphological features are valuable for the differential diagnosis of pulmonary nodules (6).

CT texture analysis can objectively and effectively evaluate the CT value of each pixel in the lesion and can detect the subtle density changes in the lesion that cannot be observed by the naked eye, reflecting to some extent the heterogeneity of the tumor (7). CT texture analysis has now been shown to distinguish between tumor grade and genetic mutations (8–10). Digumarthy et al. (11) performed CT texture analysis in 175 patients with pulmonary nodules prior to operation and showed that CT texture analysis could reliably predict well-differentiated and poorly differentiated pulmonary malignant tumors. Awe et al. (12) analyzed the application of CT texture analysis in pancreatic lesions, showing the clinical potential of CT texture analysis in the diagnosis and risk classification of pancreatic lesions. Despite the usefulness of CT texture analysis in tumor diagnosis and grading, results have been obtained in the decision-making and efficacy assessment of treatment options, but the lack of uniform standards for image texture feature parameters has led to inconsistent conclusions (7).

The hallmarks of cancer were reported by Robert Weinberg and Douglas Hanahan in 2000 (13, 14), which can intervene in tumor stages. The main reason for this is abnormal cellular energy metabolism. Cell energy metabolism technology has played an important role in research recently, which quantitatively and automatically reflects the status of the living cells, such as mitochondrial oxygen consumption rate and glycolytic acid production rate. Some earlier proteomic studies in lung cancer diagnosis based on urine or serum specimens have been investigated. Prospective biomarker studies have shown elevated DNA methylation markers CDO1 and SOX17 in the urine of patients with non-small-cell lung cancer (NSCLC) (15). Another prospective study showed that an untargeted urinary metabolome was associated with a lower lung cancer risk in never-smoking women and suggested that an abnormal urine metabolome may be associated with a higher risk of lung cancer (16). However, few studies have focused on the role of the urocyte energy metabolome in the discrimination between benign and malignant nodules.

In this study, morphological assessment, CT texture analysis, and urine cell energy metabolism test were used to investigate their values in the diagnosis of benign and malignant pulmonary nodules and to compare the diagnostic effectiveness of each feature alone and in combination.

## Materials and methods

### Ethical approval of the study protocol

The protocol of this prospective study was approved by the ethics committee of Zhongshan Hospital Affiliated to Dalian University (No. 2021029, Dalian, China). Informed consent was obtained from each patient.

### Patients and study design

**Figure 1:**
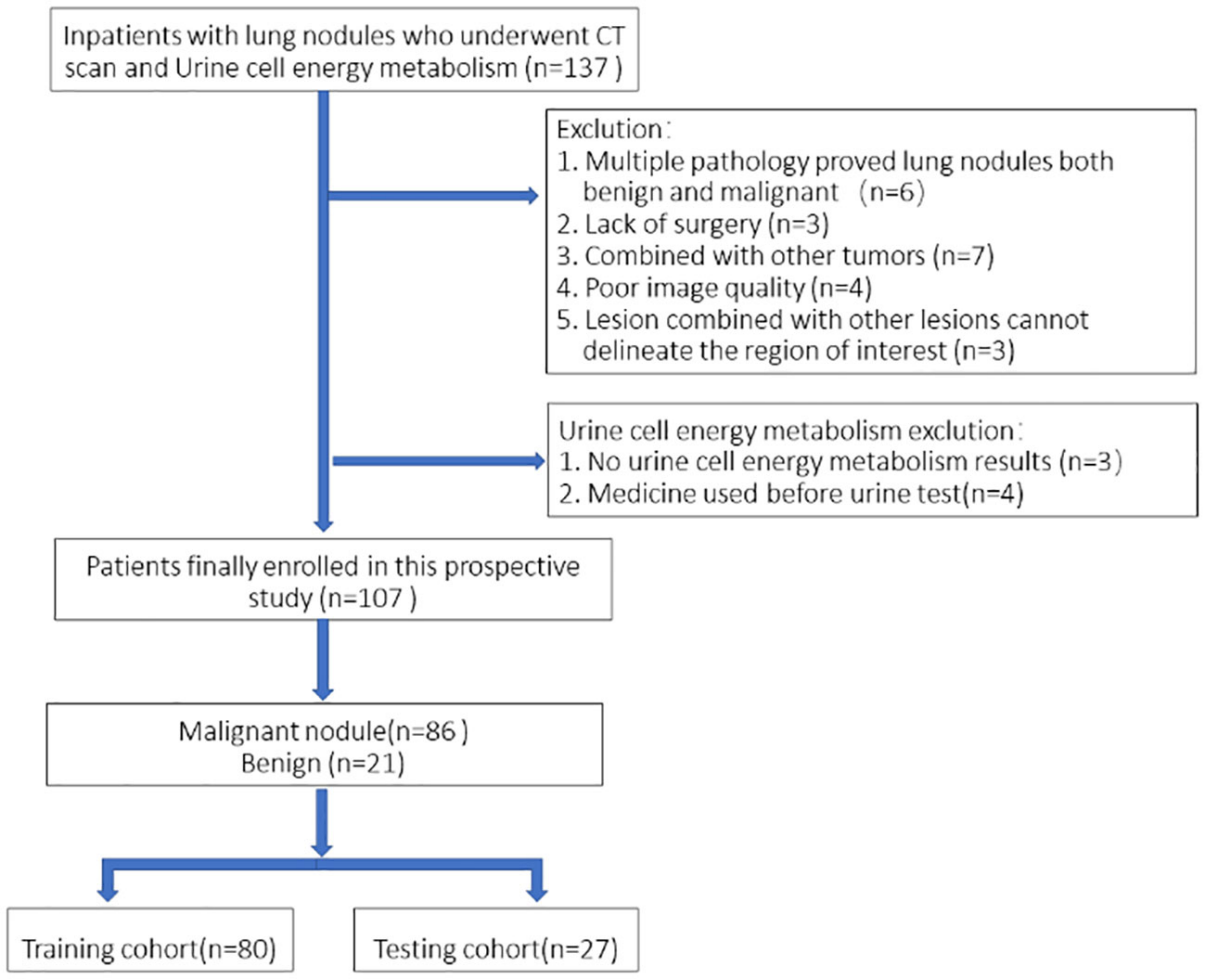
Flowchart of the study subjects based on exclusion criteria.

This was a single-institution prospective study with 107 patients eventually enrolled consecutively and urine collected from September 2021 to August 2022 at the inpatient Thoracic Surgery Department. All of the patients underwent a chest CT scan within 7 days prior to surgery and were registered as patients with a single pulmonary nodule. All patients received pulmonary surgery (video-assisted thoracoscopic surgery), and pathology results were obtained. Urine energy metabolism index was performed on all patients (Dalian DeTecsen Biomedical Co., Ltd., Dalian, China). Inclusion criteria: 1) All patients received plain CT scans and urine energy level tests in our hospital before surgery; 2) Postoperative pathological results were determined. Exclusion criteria: 1) Patients with multiple nodules including pathologically confirmed benign and malignant lesions; 2) Poor image quality due to respiratory and motion artifacts during scanning; 3) Lesions with other lesions that do not properly depict the region of interest (ROI). A flowchart of the study subjects is shown in **Figure 1**.

### CT scanning techniques

The patient was scanned in the supine position after deep inspiration in a breath-hold position. The scanning area was from the apex of the lung to the level of the bilateral costophrenic angle (including the whole lung). Siemens Somatom FLASH scanner was used for CT scanning, with a tube voltage of 120 kV, tube current automatic mAs technology, the pitch of 1.0, matrix of 512 × 512, a layer thickness of 1.0 mm, and the bone reconstruction algorithm.

### Urine cell energy metabolism test

The urine cell energy metabolism index was tested on all of the patients (Dalian DeTecsen Biomedical Co., Ltd., Dalian, China). Morning urine was collected within 3 days before surgery, and all of the patients were asked not to eat or drink for more than 8 h. By using enzymes and cofactors, the cellular energy metabolites and their derivatives in the urine cell energy metabolism can reflect a stable color reaction with the probe. The qualitative results can be obtained by colorimetric measurement at 450 nm wavelength. According to the color reaction, urine energy metabolism results were classified into four degrees: negative (0), weak positive (1), positive (2), and strong positive (3).

### Data preprocessing and analysis

In this work, 107 patients were enrolled; 80 cases were randomly selected as the training cohort and 27 patients as the testing cohort. Clinical features including urine energy metabolism results, age, gender, CT values, nodule diameter, and edge and nodule position were collected. Images of all nodules were independently segmented by two radiologists and measured using a double-blind method. Calculation of intraclass correlation coefficient (ICC) ≥0.75 was considered robust. All of the features were divided into three groups: (I) geometry, (II) intensity, and (III) texture. Geometric features characterize the three-dimensional shape of the tumor. The intensity features describe the voxel intensities within the tumor. The texture features describe the patterns and higher-order spatial distributions of the intensities.

### Feature extraction, selection, and model building

All radiomic features were extracted using Pyradiomic’s in-house feature analysis program (http://pyradiomics.readthedocs.io). Several different texture feature extraction methods were used, including the gray-level run length matrix (GLRLM), gray-level size zone matrix (GLSZM), gray-level co-occurrence matrix (GLCM), and neighborhood gray-tone difference matrix (NGTDM) methods. The least absolute shrinkage and selection operator (LASSO) regression model was used for signature construction of the discovery dataset. After LASSO feature screening, the final retained features are put into machine learning models, including logistic regression (LR), support vector machine (SVM), random forest, and XGBoost, for risk model construction.

**Figure 2:**
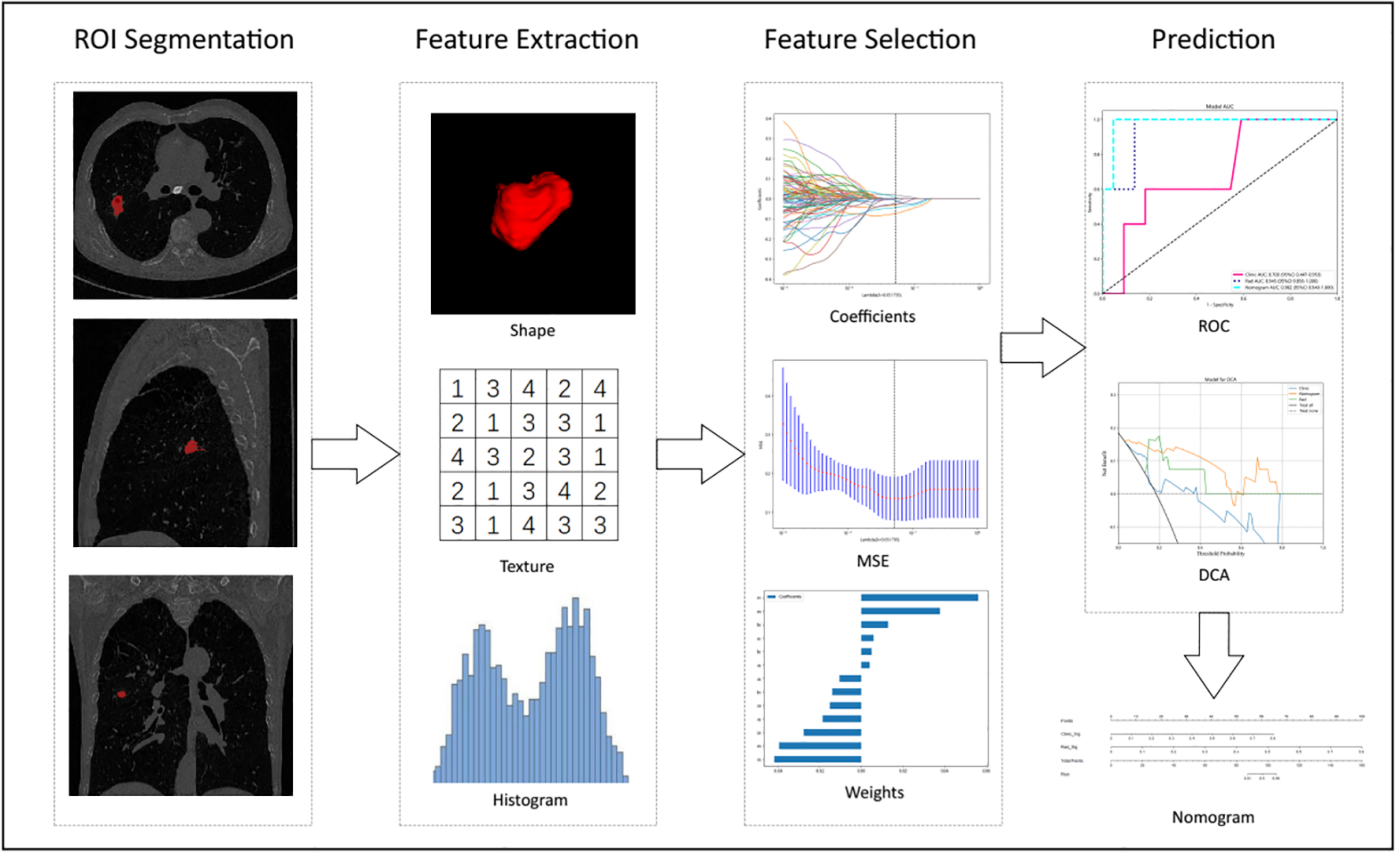
Workflow of radiomic analysis in this study. Nodules were segmented by radiologists, and features were extracted and selected by LASSO based on which the prediction model, DCA, Decision curve analysis, and nomogram were built.

Radiomic features were constructed from correlation filters, and the most robust non-redundant and predictive features were selected by LASSO. Finally, a combined nomogram model was built with clinical signatures and radiomic signatures for final interpretation and analysis. **Figure 2** shows the workflow of radiomic analysis in this study. To reduce the side effects of outliers, all pixel values were sorted for each image and truncation with an intensity range of 0.5–99.5 percentiles. Spatial normalization was employed to reduce the voxel spacing variation effect. A fixed resolution resampling method was used in our experiment to handle the aforementioned problems.

The radiomic nomogram was established in combination with radiomic signature and clinical signatures. The diagnostic efficacy of the radiomic nomogram was tested in the test cohort, and receiver operating characteristic (ROC) curves were drawn to evaluate the diagnostic efficacy of the nomogram. The calibration efficiency of the nomogram was evaluated by plotting calibration curves, and Hosmer–Lemeshow analytical fit was used to evaluate the calibration ability of the nomogram. Mapping decision curve analysis (DCA) was adopted to evaluate the clinical utility of the predictive models.

### Statistics

Clinical features, including urine energy metabolism results, age, gender, CT values, nodule diameter, and edge and nodule position, were analyzed by t-test, Mann–Whitney U test, or χ^2^ test. *P* values<0.05 were significantly considered and ultimately used. For the repeatability of the features, Spearman’s rank correlation coefficient was also used to calculate the correlation between features and to retain one of the features with a correlation coefficient >0.9 between any two features. To maximize the ability to retain the depicted features, we used a greedy recursive deletion strategy for feature filtering, that is, the feature with the greatest redundancy in the current set is removed each time. The LASSO regression model was used for the signature-constructed discovery dataset. Subsequently, we obtained a radiomic score for each patient by retaining a linear combination of features, weighted by their model coefficients. The Python scikit-learn package was used for LASSO regression modeling.

## Results

### Comparison of patient clinical characteristics

A total of 107 cases of pulmonary nodules were analyzed and separated into malignant and benign groups according to the pathology results (80 *vs*. 27 cases). The mean age of the malignant and benign groups was 63.84 ± 9.69 years *vs*. 56.57 ± 13.16 years, respectively (*P* = 0.005). There was no significant difference in terms of gender between the two groups (*P* = 0.389). **Table 1** shows the baseline clinical characteristics of patients in our cohort. Age, long diameter, short diameter, diameter, and energy level showed significant differences (*P*< 0.05) in our cohort for clinical feature construction, but the differences between CT value, position, and edge were not significant (*P* > 0.05).

**Table 1:** Baseline clinical characteristics of patients in our cohort.

Features	All (n=107)	Malignant (n=86)	Benign (n=21)	*P* value
Age (years)	62.41±10.79	63.84±9.69	56.57±13.16	0.005
Long diameter(mm)	23.24±17.73	25.06±18.14	15.78±13.97	0.031
Short diameter(mm)	16.08±11.77	17.45±12.37	10.45±6.49	0.014
Diameter(mm)	19.66±14.54	21.26±15.07	13.11±9.95	0.021
CT value (HU)	-172.83±326.18	-154.69±333.26	-247.08±290.95	0.246
Gender				0.389
0	60 (0.561)	50 (0.581)	10 (0.476)	
1	47 (0.439)	36 (0.419)	11 (0.524)	
Position				0.222
0	27 (0.252)	24 (0.279)	3 (0.143)	
1	24 (0.224)	18 (0.209)	6 (0.286)	
2	5 (0.047)	4 (0.047)	1 (0.048)	
3	32 (0.299)	28 (0.326)	4 (0.191)	
4	19 (0.178)	12 (0.139)	7 (0.333)	
Edge				0.744
0	39 (0.365)	32 (0.372)	7 (0.333)	
1	68 (0.636)	54 (0.628)	14 (0.667)	
Urine energy metabolism				0.048
Negative(0)	27 (0.252)	17 (0.198)	10 (0.476)	
Weak positive(1)	22 (0.206)	19 (0.221)	3 (0.143)	
Positive(2)	50 (0.467)	44 (0.512)	6 (0.286)	
Strong positive(3)	8 (0.075)	6 (0.069)	2 (0.095)	

### Feature selection and Rad score establishment

**Figure 3:**
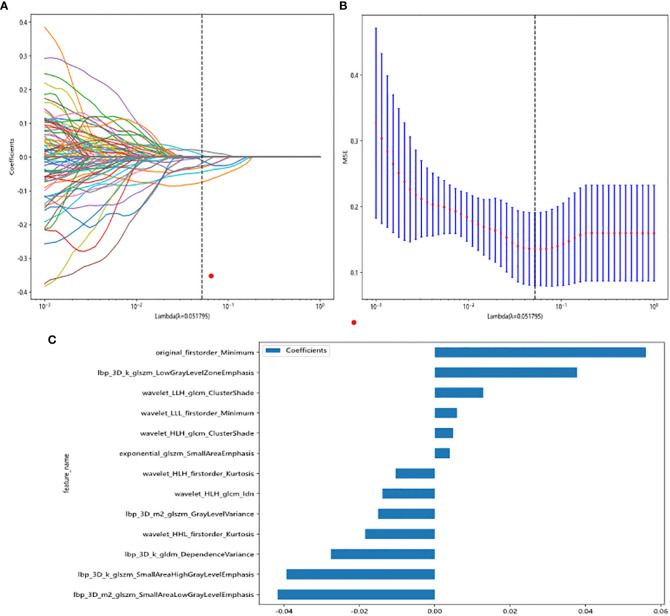
Radiomic feature selection based on LASSO algorithm and Rad score establishment. **(A, B)** Ten-fold cross-validated coefficients and 10-fold cross-validated MSE. **(C)** The histogram of the Rad score based on the selected features.

All radiomic features were extracted, and prediction models were constructed using the selected features. A total of 13 features with non-zero coefficients were selected to establish the Rad score with a LASSO LR model. **Figure 3** shows the coefficients and mean standard error (MSE) for the 10-fold validation. **Figure 3C** shows the coefficient values for the final selection of non-zero features.

Rad score is shown as follows:


$$\begin{array}{l} Rad\_score=0.21873191752687882+0.003986*\\ \exp onential\_glszm\_SmallAreaEmphasis\\ -0.027538*lbp\_3D\_k\_gldm\_DependenceVariance+\\ 0.037759*lbp\_3D\_k\_glszm\_LowGrayLevelZoneEmphasis-\\ 0.039335*lbp\_3D\_k\_glszm\_SmallAreaHighLevelEmphasis-\\ 0.015027*lbp\_3D\_m2\_glszm\_GrayLevelVariance-\\ 0.041688*lbp\_3D\_m2\_glszm\_SmallAreaLowGrayLevel\\ Emphasis+0.056017*original\_firstorder\_Minimum-\\ 0.018478*wavelet\_HHL\_firstorder\_Kurtosis-\\ 0.010406*wavelet\_HLH\_firstorder\_Kurtosis+\\ 0.004868*wavelet\_HLH\_glcm\_ClusterShade-\\ 0.013882*wavelet\_HLH\_glcm\_Idn+\\ 0.012870*wavelet\_LLH\_glcm\_ClusterShade\\ +0.005921*wavelet\_LLL\_firstorder\_Minimum \end{array}$$

**Figure 4:**
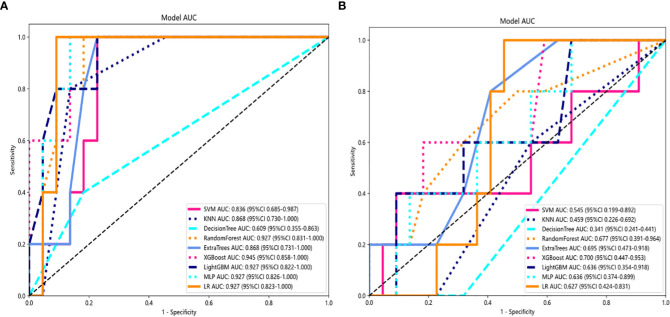
Comparison of radiometric feature model predictions for the training **(A)** and testing cohorts **(B)**. XGBoost achieved the best performance in both the training and testing cohorts.

Several models were built and compared to find the best performing model. **Supplementary Table S1** shows all of the models we used to predict benign and malignant pulmonary nodules, and the XGBoost model had the best performance compared to the other models. XGBoost achieved the best value of area under the curve (AUC) in the training and testing cohorts, with AUCs of 0.999 and 0.945 for predicting benign and malignant lung nodules, respectively. Therefore, when building clinical features, XGBoost was chosen as the base model. The optimal model was obtained by comparing the radiomic features with LR, SVM, k-nearest neighbor (KNN), decision tree, random forest, extra trees, XGBoost, and lightGBM classifier. **Figure 4** showed the ROC analysis of different models on radiomic signatures.

### Comparison of clinical, radiomic, and nomogram models

**Figure 5:**
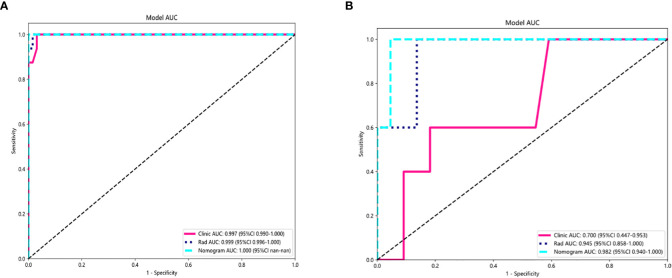
AUC Comparison of clinical, radiological, and nomogram models in the training **(A)** and testing **(B)** cohorts. The combined nomogram performed optimally in both the training and testing cohorts.

For the AUC, the clinical features [0.997, 95% confidence interval (CI) = 0.990–1.000) and the radiomic features (0.999, 95% CI = 0.996–1.000) were perfectly fitted for the training cohort. In the testing cohort, the clinical characteristics appeared to be overfitted (0.700, 95% CI = 0.473–0.953), but the radiomic signature remained well fitted (0.945, 95% CI = 0.858–1.000). The nomogram using the LR algorithm, combining clinical features and radiomic features, showed the best performance (0.982, 95% CI = 0.940–1.000). In order to compare the clinical signature and radiomic signature and nomogram, DeLong test was used. The results indicated that the AUC comparison between the nomogram and the clinical signature achieved 0.019 and that the nomogram model outperformed the clinical model in the discrimination between malignant and benign nodules. The AUC comparison between the nomogram and radiomic achieved 0.457, which means that both models performed well in differentiating malignant and benign nodules. **Figure 5** showed the AUC in both the training and testing cohorts.

**Figure 6:**
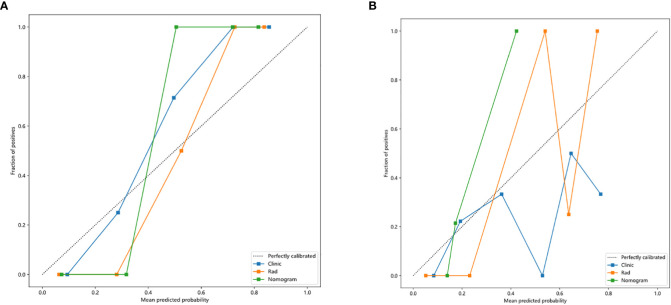
Calibration curves in the training and testing cohorts showing that the nomogram fits perfectly well in both the training **(A)** and testing cohorts **(B)**.

Nomogram calibration curves showed good agreement between predicted and observed benign and malignant pulmonary nodules in the training and testing cohorts. The *P* values for the Hosmer–Lemeshow test were 0.109, 0.832, and 0.123 in respect of clinical signature, radiomic signature, and nomogram, suggesting that the nomogram fitted perfectly in both the training and testing cohorts. **Figure 6** showed the calibration curves in the training and testing cohorts.

**Figure 7:**
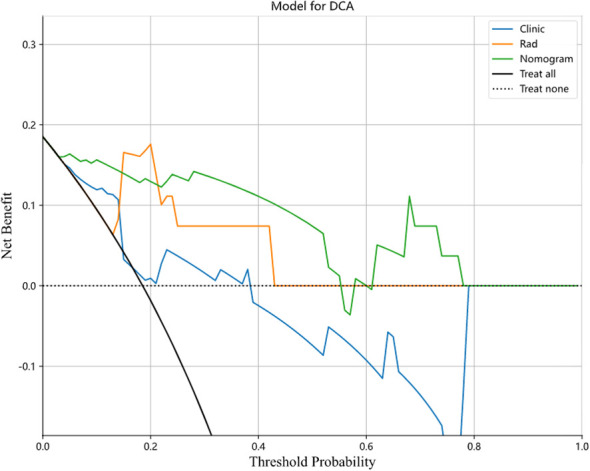
Decision curves of the clinical, radiomic, and nomogram models in the testing cohort. Nomogram model shows the best clinical benefit in predicting benign and malignant lung nodules compared to the clinical and radiological models.

**Figure 8:**
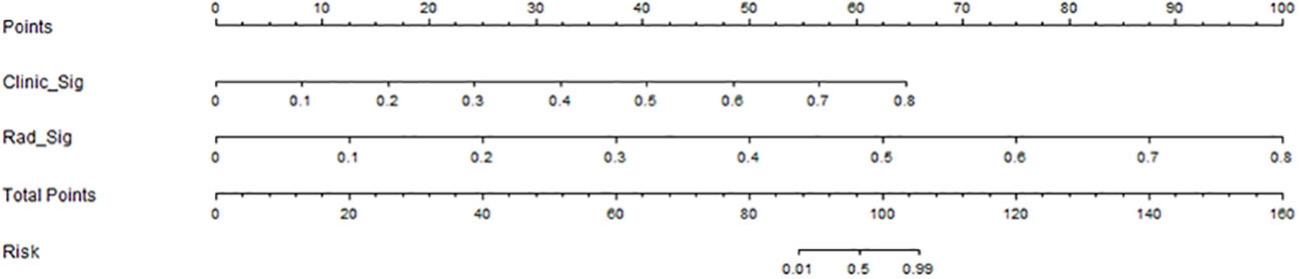
Clinical application of the nomogram in the differentiation of benign and malignant pulmonary nodules.

In this study, we also evaluated each model through the DCA. The DCA for the clinical features, radiomic features, and radiomic nomogram is presented in **Figure 7**. Compared to the scenario without the prediction model (i.e., all treatment or no treatment regimen), the radiomic nomogram significantly improved the patient’s intervention outcome with a prediction probability of 0.05–0.78 compared to 0.05–0.38 for the clinical features and 0.12–0.43 for the radiomic signature. Nomograms were higher than other signatures. The preoperative use of radiological nomograms to predict benign and malignant pulmonary nodules showed better clinical benefit. **Figure 8** shows the nomogram for clinical use, with the total score reflecting the probability of malignancy in pulmonary nodules.

## Discussion

This study showed that for the diagnosis of pulmonary nodules, the combined model based on radiomic features and clinical features including urine energy level had higher diagnostic performance than the radiomic features and clinical features alone. The prediction probability was higher than that of a single method, suggesting that the combination of the two methods is more advantageous in identifying benign and malignant pulmonary nodules.

Radiomics aims to develop new imaging biomarkers to better understand the microbiology of cancer (17) and to provide additional data on the biological composition of lung nodules, which is frequently used for lung cancer screening and diagnosis. Multiple studies have demonstrated the effectiveness of radiomics in discriminating between malignant and benign nodules. Our study showed that the radiomic features performed better than clinical features in both the training cohort and testing cohort. Several radiomic features contribute to the identification of malignant nodules, such as kurtosis and entropy, which have a sensitivity of 83% and specificity of 69% for assessing lung nodule identification. These findings were also reported in previous studies by Sacconi et al. (9), and these CT texture parameters (e.g., skewness and entropy) are also good predictors of epidermal growth factor receptor (EGFR) mutations and lung adenocarcinoma patient survival. Several studies have demonstrated that radiomics was an effective tool in differentiating between malignant and benign tumors, with an accuracy of 79.06%–81%, a sensitivity of 76.2%–78.00%, and a specificity of 76.11%–91.7% (18, 19). Another study (20) showed that radiomic signatures achieve an AUC of 72% for the classification of malignant and benign nodules but with limited accuracy (11). Radiomic texture analysis and CT features are more effective in distinguishing persistent pulmonary nodules from transient pulmonary tuberculosis than clinical and CT features alone (21). In addition, it is difficult to distinguish the invasion degree of lung adenocarcinoma only by traditional CT features alone (22, 23). This was consistent with the study by Kumar et al. (18), which showed that the accuracy of differentiation between malignant and benign nodules reached 79.06%, with a sensitivity of 78.00% and specificity of 76.11%. In the study by Wu et al. (20), radiomic signature allowed the classification of malignant and benign nodules with an AUC equal to 72%.

In order to more accurately identify the macroscopic and microscopic changes of lung nodules and comprehensively demonstrate the changes of tumor heterogeneity in lung nodules, the combined prediction method can not only absorb the morphological changes of lung nodules but also reflect the characteristics of the microstructure of lung nodules in combination with radiomic features (24). In our investigation, the clinical signature (0.997, 95% CI = 0.990–1.000) and radiomic signature (0.999, 95% CI = 0.996–1.000) both achieve the ideal fitting in the training cohort. Clinical signatures in the testing cohort appear to be overfitting those who attained 0.700, 95% CI = 0.473–0.953, although radiomic signatures continued to match well (0.982, 95% CI = 0.940–1.000). Several studies have tried to compare the added value of clinical features with these radiomic features. In fact, they could improve the performance of machine learning methods to differentiate between focal pneumonia and adenocarcinoma (25) or NSCLC (26). The addition of clinical features could not also produce an improvement in the model performance (27), highlighting the importance of the radiomic features. In nearly all cases, the diagnostic accuracy is improved by combining the radiomic model with clinical data, such as serum markers, demographics, histopathology, and genomics (28). These results were consistent with our results that the combined nomogram model based on radiomic and clinical features performed best in the differentiation of malignant and benign nodules.

Urinary tests had been used as noninvasive cost-effective tools for cancer detection (29), the components of which can reflect the circulome of the tumor. Studies have shown that urine can indicate lung cancer by proteomic biomarker panels (30). Urine cellular energy metabolism as a body fluid for lung nodule diagnosis has several advantages. First, it can be easily obtained. Second, urinary metabolism index was reliably detected by mass spectrometry (MS) (31–33). Studies (34–37) have shown significant differences between patients with lung cancer and healthy subjects based on urine metabolomic profiles. A cross-validated model based on nuclear magnetic resonance (NMR) spectroscopy differentiated lung cancer (n = 71) from healthy controls (n = 54) with a sensitivity and specificity of 93% and 94%, respectively (35). Our result showed that the combined model including clinical features and urine energy metabolism index showed the best predicting performance. This is consistent with the opinion of Zhang et al. (38) that urinary biomarkers help discriminate lung cancer from control groups, which may be an auxiliary diagnostic tool for lung cancer detection along with radiology features. Considering the complexity of the pathways and metabolites in the disease processes, many biological explanations are hypothetical and unsupported by evidence. Metabolites may increase during the initial stages of the disease process but decrease rapidly as the disease progresses (29). The urinary metabolomic test has promising clinical utility; these studies still need additional distinct validation as the next step toward clinical implementation.

This study has the following limitations: 1) the sample size is small, and there may be selection bias; 2) The boundary of lesions was manually delineated, and some small blood vessels or bronchi may not be completely avoided, and human error is unavoidable. In conclusion, radiomic analysis of pulmonary nodules and clinical features including urine energy levels are valuable for the differential diagnosis of benign and malignant pulmonary nodules, and their combined model has a high diagnostic efficiency.

## Conclusions

The combined nomogram model based on radiomic and clinical signature-urine including cellular energy features is helpful for the prediction of benign and malignant pulmonary nodules. The model has higher predictive performance compared with models based on radiomic and clinical features only and is expected to provide more information for future decisions on pulmonary nodules.

## Data availability statement

The raw data supporting the conclusions of this article will be made available by the authors, without undue reservation.

## Ethics statement

The studies involving human participants were reviewed and approved by Affiliated Zhongshan Hospital of Dalian University. The patients/participants provided their written informed consent to participate in this study. Written informed consent was obtained from the individual(s) for the publication of any potentially identifiable images or data included in this article.

## Author contributions

JS, JW and XZ conceived the study. JS, LW, LD and DY collected the data. JS, YW, RZ and HD analyzed the data. JS wrote the manuscript. JW and XZ provided study supervision. All authors contributed to the article and approved the submitted version.
