# Supplementary material for: A novel nomogram model combining CT texture features and urine energy metabolism to differentiate single benign from malignant pulmonary nodule
Source: Front Oncol. 2022 Dec 15;12:1035307. doi: 10.3389/fonc.2022.1035307 (PMC9798090; doi:10.3389/fonc.2022.1035307)
Supplement: Supplementary file 1 [file Table_1.docx]

Supplementary Table S1：All models used for benign and malignant pulmonary nodule prediction.

| Model_  name | Accuracy | AUC | 95% CI | Sensitivity | Specificity | PPV | NPV | Precision | Recall | F1 | Threshold | Task |
| --- | --- | --- | --- | --- | --- | --- | --- | --- | --- | --- | --- | --- |
| SVM | 0.975 | 0.968 | 0.906- 1.000 | 0.938 | 0.984 | 0.938 | 0.984 | 0.938 | 0.938 | 0.938 | 0.188 | label-train |
| SVM | 0.815 | 0.836 | 0.685- 0.988 | 1.000 | 0.809 | 0.500 | 1.000 | 0.500 | 1.000 | 0.667 | 0.221 | label-test |
| KNN | 0.863 | 0.883 | 0.812- 0.954 | 0.750 | 0.905 | 0.632 | 0.934 | 0.632 | 0.750 | 0.686 | 0.400 | label-train |
| KNN | 0.852 | 0.868 | 0.729- 1.000 | 0.800 | 0.905 | 0.571 | 0.950 | 0.571 | 0.800 | 0.667 | 0.400 | label-test |
| Decision  Tree | 1.000 | 1.000 | nan- nan | 1.000 | 1.000 | 1.000 | 1.000 | 1.000 | 1.000 | 1.000 | 1.000 | label-train |
| Decision  Tree | 0.741 | 0.609 | 0.355- 0.863 | 0.400 | 1.000 | 0.333 | 0.857 | 0.333 | 0.400 | 0.364 | 1.000 | label-test |
| Random  Forest | 0.988 | 0.996 | 0.986- 1.000 | 1.000 | 0.984 | 0.941 | 1.000 | 0.941 | 1.000 | 0.969 | 0.400 | label-train |
| Random  Forest | 0.852 | 0.927 | 0.831- 1.000 | 1.000 | 0.818 | 0.556 | 1.000 | 0.556 | 1.000 | 0.714 | 0.400 | label-test |
| Extra  Trees | 1.000 | 1.000 | nan- nan | 1.000 | 1.000 | 1.000 | 1.000 | 1.000 | 1.000 | 1.000 | 1.000 | label-train |
| Extra  Trees | 0.815 | 0.868 | 0.731- 1.000 | 1.000 | 0.773 | 0.500 | 1.000 | 0.500 | 1.000 | 0.667 | 0.300 | label-test |
| XG  Boost | 0.988 | 0.999 | 0.996- 1.000 | 1.000 | 0.984 | 0.941 | 1.000 | 0.941 | 1.000 | 0.969 | 0.457 | label-train |
| XG  Boost | 0.889 | 0.945 | 0.858-1.000 | 1.000 | 0.864 | 0.625 | 1.000 | 0.625 | 1.000 | 0.769 | 0.522 | label-test |
| Light  GBM | 0.863 | 0.938 | 0.889- 0.988 | 1.000 | 0.841 | 0.593 | 1.000 | 0.593 | 1.000 | 0.745 | 0.250 | label-train |
| Light  GBM | 0.815 | 0.927 | 0.822 - 1.000 | 1.000 | 0.773 | 0.500 | 1.000 | 0.500 | 1.000 | 0.667 | 0.287 | label-test |
| MLP | 0.875 | 0.904 | 0.827- 0.982 | 0.813 | 0.891 | 0.650 | 0.950 | 0.650 | 0.813 | 0.722 | 0.296 | label-train |
| MLP | 0.889 | 0.927 | 0.826- 1.000 | 1.000 | 0.905 | 0.625 | 1.000 | 0.625 | 1.000 | 0.769 | 0.326 | label-test |
| LR | 0.788 | 0.893 | 0.820- 0.965 | 0.936 | 0.750 | 0.484 | 0.979 | 0.484 | 0.938 | 0.638 | 0.151 | label-train |
| LR | 0.926 | 0.927 | 0.823- 1.000 | 1.000 | 0.952 | 0.714 | 1.000 | 0.714 | 1.000 | 0.833 | 0.525 | label-test |
